# Spatiotemporal profile of altered neural reactivity to food images in obesity: Reward system is altered automatically and predicts efficacy of weight loss intervention

**DOI:** 10.3389/fnins.2023.948063

**Published:** 2023-02-08

**Authors:** Vahe Poghosyan, Stephanos Ioannou, Khalid M. Al-Amri, Sufana A. Al-Mashhadi, Fedaa Al-Mohammed, Tahani Al-Otaibi, Wjoud Al-Saeed

**Affiliations:** ^1^Department of Neurophysiology, National Neuroscience Institute, King Fahad Medical City, Riyadh, Saudi Arabia; ^2^Department of Physiological Sciences, Alfaisal University, Riyadh, Saudi Arabia; ^3^Obesity, Endocrinology and Metabolism Center, King Fahad Medical City, Riyadh, Saudi Arabia; ^4^Research Unit, National Neuroscience Institute, King Fahad Medical City, Riyadh, Saudi Arabia

**Keywords:** obesity, magnetoencephalography (MEG), preconscious, reward system, neural reward reactivity, food image processing, predict weight loss, automatic process (vs. controlled)

## Abstract

**Introduction:**

Obesity presents a significant public health problem. Brain plays a central role in etiology and maintenance of obesity. Prior neuroimaging studies have found that individuals with obesity exhibit altered neural responses to images of food within the brain reward system and related brain networks. However, little is known about the dynamics of these neural responses or their relationship to later weight change. In particular, it is unknown if in obesity, the altered reward response to food images emerges early and automatically, or later, in the controlled stage of processing. It also remains unclear if the pretreatment reward system reactivity to food images is predictive of subsequent weight loss intervention outcome.

**Methods:**

In this study, we presented high-calorie and low-calorie food, and nonfood images to individuals with obesity, who were then prescribed lifestyle changes, and matched normal-weight controls, and examined neural reactivity using magnetoencephalography (MEG). We performed whole-brain analysis to explore and characterize large-scale dynamics of brain systems affected in obesity, and tested two specific hypotheses: (1) in obese individuals, the altered reward system reactivity to food images occurs early and automatically, and (2) pretreatment reward system reactivity predicts the outcome of lifestyle weight loss intervention, with reduced activity associated with successful weight loss.

**Results:**

We identified a distributed set of brain regions and their precise temporal dynamics that showed altered response patterns in obesity. Specifically, we found reduced neural reactivity to food images in brain networks of reward and cognitive control, and elevated reactivity in regions of attentional control and visual processing. The hypoactivity in reward system emerged early, in the automatic stage of processing (< 150 ms post-stimulus). Reduced reward and attention responsivity, and elevated neural cognitive control were predictive of weight loss after six months in treatment.

**Discussion:**

In summary, we have identified, for the first time with high temporal resolution, the large-scale dynamics of brain reactivity to food images in obese versus normal-weight individuals, and have confirmed both our hypotheses. These findings have important implications for our understanding of neurocognition and eating behavior in obesity, and can facilitate development of novel integrated treatment strategies, including tailored cognitive-behavioral and pharmacological therapies.

## 1. Introduction

Obesity is a highly prevalent condition (Chooi et al., 2019; WHO, 2020) with significant health and economic implications (Flegal et al., 2005; Wyatt et al., 2006; Finkelstein et al., 2009; Cecchini et al., 2010). Multiple factors contribute to its development and persistence; however, energy imbalance driven by overconsumption of high-calorie food is the main cause of obesity (Swinburn et al., 2009; Hall et al., 2012; Crino et al., 2015). Brain regulates appetite and food intake, and thus plays a central role in promoting and maintaining obesity (Morton et al., 2006; Rolls, 2007; Berthoud and Morrison, 2008; Berthoud et al., 2017). However, the exact neural mechanisms that support obesogenic behavior are still poorly understood.

The most prevalent neurocognitive theories of obesity have focused on brain reward system, suggesting that food-related reward reactivity is the primary driver of obesogenic behavior (Stice and Yokum, 2016; Devoto et al., 2018; Stice and Burger, 2019). According to the dynamic vulnerability model (Stice and Yokum, 2016; Stice and Burger, 2019), an initial aberrant neural reward response to high-calorie food intake, which is presumably an inborn characteristic, promotes overeating, which with time results in blunted reward response to food intake and elevated response to associated cues (e.g., images of food). Consequently, reward hyper-responsivity to food cues, which induces craving when these cues are encountered, becomes the main driver of overeating.

Other models have prioritized cognitive control network of the brain, suggesting that the capacity of this network to regulate reward system reactivity to food cues is the primary driver of eating behavior, rather than the reward system responsivity itself (Lowe et al., 2019). Thus according to this model, poor recruitment of cognitive control regions and weaker modulatory control over reward system during food cue processing leads to overeating and weight gain. Balance models on the other hand suggest that eating behavior may not result from the brain reward and cognitive control networks acting independently or in direct competition, but may result from a balance between their activities in response to food cues (Lopez et al., 2017; Devoto et al., 2022). Overeating in this case will ensue when activity balance favors reward over cognitive control network.

Neuroimaging studies supporting these models have found that food images relative to non-food stimuli activate brain regions associated with reward processing, cognitive and attentional control, and visual processing (van der Laan et al., 2011; Dagher, 2012; García-García et al., 2013; Hollmann et al., 2013; Huerta et al., 2014). Additional evidence indicates that these activations, modulated by person’s current homeostatic state (Cornier et al., 2007; Führer et al., 2008; Siep et al., 2009), various cognitive processes (Grabenhorst et al., 2008; Kober et al., 2010; Hare et al., 2011; Frankort et al., 2012; Hollmann et al., 2012; Siep et al., 2012; Silvers et al., 2014; Pohl et al., 2017; Franssen et al., 2020), perceived caloric content (Goldstone et al., 2009; Murdaugh et al., 2012; Killgore et al., 2013), and other internal and external factors (Devoto et al., 2022), affect their food perception and eating behavior. Notably, increased food-cue reactivity within the reward system has been associated with craving (Siep et al., 2012; Frankort et al., 2014; Miedl et al., 2018) and weight gain (Yokum et al., 2011, 2014; Demos et al., 2012; Stice et al., 2015), while activity within the cognitive control network has been associated with anti-obesogenic behavior (Lopez et al., 2014, 2016; Weygandt et al., 2015) and weight loss (Weygandt et al., 2013; Neseliler et al., 2019), and negatively associated with weight gain (Kishinevsky et al., 2012). Brain regions of the reward system implicated in these studies include orbitofrontal cortex (OFC), ventromedial prefrontal cortex (VMPFC), anterior cingulate cortex (ACC), striatum, insula and amygdala. Regions of the cognitive control network most commonly activated in response to food cues are inferior frontal gyrus (IFG) and dorsolateral prefrontal cortex (DLPFC).

In line with these findings, a volume of literature has shown an association between elevated weight and altered brain function. Most neuroimaging studies have found greater reward system reactivity to images of food in obese and overweight individuals compared with normal-weight controls (Rothemund et al., 2007; Stoeckel et al., 2008; Bruce et al., 2010; Martin et al., 2010; Dimitropoulos et al., 2012; Nummenmaa et al., 2012; Scharmüller et al., 2012; Pursey et al., 2014), although contrary and null results have also been reported (Frankort et al., 2012; Murdaugh et al., 2012). Furthermore, neural reactivity to food cues in the cognitive control network has been found to be reduced in obese individuals (Dimitropoulos et al., 2012; Nummenmaa et al., 2012; Brooks et al., 2013; Tuulari et al., 2015), and negatively correlated with body mass index (BMI) in food-related cognitive control tasks (Batterink et al., 2010; Giuliani et al., 2014; Janssen et al., 2017; Han et al., 2018). It is thus evident from the extant literature that altered food cue reactivities in the reward system and cognitive control network play key roles in etiology and maintenance of obesity. However, to fully understand their exact roles and the intricate neural mechanisms associated with obesity, information about temporal dynamics of these neural activations in obese and normal-weight individuals is needed, but so far remains unknown. It is also unknown if the altered responses in obesity arise in the early, automatic or late, controlled stage of information processing. This information could greatly extend and refine current neurocognitive models, which are based on findings from fMRI studies, and therefore unable to exploit the precise temporal dynamics of neural activity.

Lifestyle intervention for obesity, including reduced-calorie diet, increased physical activity and behavioral therapy (Jensen et al., 2014), is an effective and widely used weight loss strategy (Curioni and Lourenco, 2005; Douketis et al., 2005; Franz et al., 2007; Webb and Wadden, 2017). However, there is a considerable variability in treatment response across individuals (Curioni and Lourenco, 2005; MacLean et al., 2011; Twells et al., 2021). Understanding the potential predictors of success in weight loss interventions can lead to development of effective tailored treatment strategies.

Negative energy balance induced by lifestyle intervention triggers hormonal response that aims to maintain the higher body weight (Klok et al., 2007; Sumithran et al., 2011). Among other systems, appetite hormones (e.g., leptin, ghrelin) influence neural circuits that regulate eating behavior in favor of increased calorie intake (Morton et al., 2006, 2014), including modulating food cue reactivity in the reward system (Farooqi et al., 2007; Malik et al., 2008; De Silva et al., 2011; Aotani et al., 2012) and cognitive control network (Rosenbaum et al., 2008; Guthoff et al., 2010). In addition to circulating levels of appetite hormones, genetic makeup (Stice et al., 2008, 2010, 2015; Yeo and Heisler, 2012; Lamiquiz-Moneo et al., 2019), personality traits (Grimm et al., 2012; Van Der Laan and Smeets, 2015; Vainik et al., 2019; Nakamura and Koike, 2021), cognitive function (Siep et al., 2012; Weygandt et al., 2013, 2015; Dalle Grave et al., 2014; Gettens and Gorin, 2017) and environment (Malik et al., 2011; Gorin et al., 2013; Blechert et al., 2016; Lowe et al., 2018) can modulate brain activity and affect eating behavior. Thus body weight regulation is a complex multifactorial process where neural systems that control appetite and eating behavior play a main role (Morton et al., 2006, 2014; Berthoud et al., 2017; Makaronidis and Batterham, 2018). In line with this, several longitudinal studies have found that increased neural activity in cognitive control regions during food cue processing predicts success of reduced-calorie diet (Weygandt et al., 2013, 2015), and that this activity, rather than appetite hormones, plays a critical role in weight loss (Neseliler et al., 2019).

Few studies have investigated whether reward system reactivity to food cues can predict the outcome of lifestyle intervention, but with equivocal results (Murdaugh et al., 2012; Hermann et al., 2019). Murdaugh et al. (2012) has found a significant association between high levels of pretreatment reactivity to high-calorie food images in striatum, ACC and insula of the brain’s reward system and poorer outcome in a 12-week lifestyle weight management program. In contrast, Hermann et al. (2019) failed to find a significant relationship between pretreatment neural response to high-calorie food images and outcome in a 6-month weight loss intervention; although they found that changes in food cue reactivity in striatum between pretreatment and 1 month in the treatment program predicted the 6-month outcome. Thus the utility of pretreatment neural reward reactivity to food cues in predicting the outcome of weight loss intervention is still unclear. Few prevalent models, such as the incentive sensitization and dynamic vulnerability models (Stice and Yokum, 2016; Stice and Burger, 2019), have emphasized association between elevated reward responsivity to food cues and future weight gain; however, explicit predictions regarding association of reward system reactivity and voluntary weight loss are missing from the current models.

The main objectives of the present study are to investigate the temporal dynamics of food cue reactivity in the reward system as well as other brain regions in obese versus normal-weight individuals, and examine the relationship of this reactivity to future weight change. More specifically, we aim to determine whether the altered neural reward response to images of food in obesity emerges in the early, automatic or late, controlled stage of information processing, and whether pretreatment levels of this reactivity may predict the outcome of the lifestyle weight loss intervention. Therefore, we propose two hypotheses: First, we hypothesize that neural reward response to images of food is altered in obesity beginning from the early, automatic stage of information processing. This hypothesis is based on the observations that reward-related neural signals can emerge in the brain as early as 100–150 ms after stimulus onset (Doñamayor et al., 2012; Thomas et al., 2013; Bach et al., 2017) and that obese individuals exhibit an automatic attentional bias toward food cues (Castellanos et al., 2009; Nijs et al., 2010a; Hume et al., 2015). Second, we hypothesize that the pretreatment magnitude of food cue reactivity in the reward system can predict the outcome of lifestyle intervention. Considering that increased neural food reward activity is associated with craving (Siep et al., 2012; Frankort et al., 2014; Miedl et al., 2018) and weight gain (Yokum et al., 2011, 2014; Demos et al., 2012; Stice et al., 2015), and that thinking about health consequences of food and conscious suppression of craving decreases this activity (Siep et al., 2012), we expect that reduced food cue reactivity in the reward system will be associated with successful weight loss. Further, we aim to perform whole-brain analysis to explore the dynamics of other brain systems affected in obesity. Characterization of the large-scale dynamics of brain activity during food cue processing and validations of our hypotheses will significantly improve our understanding of the neural mechanisms associated with obesity, and may have important implications for the development of appropriate intervention strategies.

Conscious access to visual sensory information occurs around 200 ms after stimulus onset (Koivisto and Grassini, 2016; Förster et al., 2020) or even later (Dehaene and Changeux, 2011; Sergent, 2018). In this study, we consider the neural responses occurring before 150 ms post-stimulus to reflect preconscious automatic information processing. To achieve our objectives, including investigating the neural temporal dynamics of food cue reactivity and differentiating the early, automatic and late, controlled brain processes, we exploit high temporal resolution and whole-brain coverage of magnetoencephalography (MEG) and distributed source imaging (Ioannides, 2006; Baillet, 2017).

## 2. Materials and methods

### 2.1. Participants

Twenty-four patients with class 2 or 3 obesity who intended to lose weight (18 women; BMI: range 35–66.7, mean [*M*] = 44.8, standard deviation [SD] = 8.1 kg/m^2^; age: *M* = 40.3, SD = 11.1 years) and 24 age- and gender-matched normal-weight controls (18 women; BMI: range 18.6–25, *M* = 22.6, SD = 2.3 kg/m^2^; age: *M* = 40, SD = 8.9 years) participated in the present study. All participants were right-handed, had normal or corrected-to-normal visual acuity, and had no history of mental or neurological disorder, diabetes, substance abuse or addiction, or serious medical illness. Patients were recruited from the outpatient clinic at the obesity, endocrine, and metabolism center of King Fahad Medical City (KFMC). Controls were recruited through hospital announcements and social media channels.

The study was approved by KFMC ethics committee. Written informed consent was obtained from all participants prior to the study.

### 2.2. Weight loss intervention

All patients were prescribed lifestyle changes, including a low-calorie diet with a recommended consumption of 1500 kcal/day and moderate physical activity of at least 180 min/week, and were counseled about these topics on individual basis. They were then followed up as deemed medically necessary (typically every 6 months).

Patients’ motivations and potential obstacles to weight loss were examined and addressed as needed. They were educated about low-calorie dieting, and were encouraged to voluntarily reduce their intake by 500 kcal/day to reach 1500 kcal/day. Patients were asked to keep detailed food diaries, including the names, types and specifications (fat, protein, carbohydrates etc.) of the consumed food, places of consumption (home, restaurant etc.) and portion sizes. They were also asked to note their thoughts on how the lifestyle changes and gained knowledge influenced their eating behavior, attitudes and understanding of nutrition. In the follow-up visits, the implementation of the recommended lifestyle changes was discussed and the experienced difficulties were examined and addressed. Further dietary and exercise counseling was given on individual basis.

### 2.3. MEG experiment

One day before the experiment, the participants were instructed to fast overnight (only drinking water was allowed). The experiments started between 8.00 and 10.00 am. Hunger enhances neural reward response (Gottfried et al., 2003; Führer et al., 2008; Siep et al., 2009; Berridge et al., 2010) and visual attention to food cues (Stockburger et al., 2009; Loeber et al., 2013; Kumar et al., 2016). Therefore, as well as for standardization purposes, we assessed our study participants in fasted state, similar to a number of previous studies, e.g., (Stoeckel et al., 2008; Murdaugh et al., 2012; Mokhtari et al., 2018).

**FIGURE 1:**
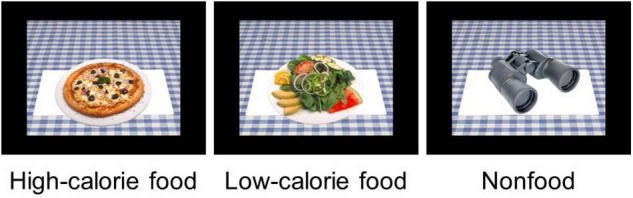
Stimuli. Examples of food and nonfood images presented to the subjects. Taken from Führer et al. (2008).

Each participant was scanned with MEG at rest and in two experimental runs. In resting-state, spontaneous brain activity was recorded for 10 min during which participants were comfortably seated in the MEG scanner with eyes open and no stimuli presented. In the experimental runs, participants were presented with images of high-calorie food, low-calorie food and nonfood items (Figure 1; Führer et al., 2008). High-calorie food images included fatty and carbohydrate-rich items, such as fast food hamburger and cake. Low-calorie food images included healthy low-fat items, such as fresh salads and fruits. Food images were categorized into high- and low-calorie by a clinical dietician based on McCance and Widdowson (2014) and Nutrinics software.^1^ Nonfood images included tools and everyday items that were clearly unrelated to food, such as watch and key. All presented food, as well as nonfood, images were of common items that are widely available in Saudi Arabia and were very familiar to participants. All pictures were matched for luminance, resolution and size, and had identical background and top-view angle, which one would typically obtain by sitting at a table.

Each run included 90 experimental trials in which 30 images from each of the three stimulus categories (high-calorie food, low-calorie food and nonfood) were presented to participants in a random order. To ensure that participants were alert and focused on visual stimuli, 13 attention-check trials, randomly interspersed among the experimental trials, were added. In these trials, a finger click icon image was presented, and participants were instructed to respond by pressing a button with the right index finger. The run was repeated if the participant missed two consecutive button presses. All images in experimental and attention-check trials were presented on a homogenous gray background on a screen located 1 m in front of the participant, with a duration of 2 s and interstimulus interval varied randomly between 2.8 and 3.2 s. Overall, across two experimental runs, 206 trials (180 experimental and 26 attention-check) were recorded in each participant, with 60 trials for each image category. Attention-check trials were excluded from the data analysis.

After the experiment, participants were shown all the food images on the computer screen or printed paper and were asked to select five foods in the order of preference that they would most likely have at that time. Additionally, they were asked to choose a portion that best fits their needs, which could be quarter, half, full, or more than one portion. This post-experimental inquiry was made in an attempt to assess each participant’s current preference for high- or low-calorie food (however, see the section “4.3. Limitations”).

### 2.4. Data acquisition and preprocessing

Participants’ weights and heights were measured before the MEG experiment and approximately 6 months after it, during the scheduled follow-up at the obesity clinic.

Magnetoencephalography data were acquired using 306-sensor (102 magnetometers and 204 planar gradiometers) whole-head Vectorview system (MEGIN/Elekta Oy, Helsinki, Finland), inside a magnetically shielded room, at a sampling rate of 2000 Hz and band-pass of 0.3–660 Hz. Simultaneously and in synchrony with MEG, electrocardiogram (ECG) and electrooculogram (EOG) were recorded to eliminate the corresponding artifacts from the MEG signals.

Raw MEG signals were visually inspected to identify noisy channels and time segments. Temporally extended signal space separation and movement correction algorithms, implemented in vendor’s Maxfilter software (MEGIN/Elekta Oy, Helsinki, Finland), were applied to suppress external and internal interferences, and compensate for noisy channels and subject’s head movement during the recording (Taulu et al., 2004; Taulu and Simola, 2006).

Subsequent data preprocessing and analysis were performed with Brainstorm (Tadel et al., 2011), which is freely available online.^2^ We followed in general the group analysis pipeline described in Tadel et al. (2019) and used several in-house developed Brainstorm plugins and scripts. MEG signals were low-pass filtered at 250 Hz, notch filtered at 60 Hz and its harmonics, and resampled to 1000 Hz. Independent component analysis (ICA), together with ECG and EOG, and followed by visual inspection of the identified independent components in spatial and temporal domains, were used to eliminate the eye and cardiac artifacts from the MEG data (Makeig et al., 1996; Lee et al., 1999). The preprocessed clean MEG data were divided into epochs, from −500 to 1000 ms with respect to stimulus onset, and were averaged separately for each stimulus category (high-calorie food, low-calorie food and nonfood) in each run.

### 2.5. MEG source analysis

For each participant, a pseudo-individual anatomy was created by warping the ICBM152 MRI template (Fonov et al., 2009) to the individual digitized head shape (more than 1,000 points). The lead fields for each run were computed using the overlapping spheres method (Huang et al., 1999) with the original sensor positions, and a volumetric grid source space with 5 mm isotropic resolution. The noise covariance was estimated from the 10-min resting-state MEG data. Weighted minimum norm estimate (MNE) with Brainstorm’s default parameter settings was used for solving the inverse problem (Baillet et al., 2001). MNE estimates the amplitude of neuroelectric currents at each point in the source grid, producing a brain activation map at 5 mm isotropic resolution.

The brain activation maps were averaged across the two experimental runs for each subject and stimulus category weighted by the number of good trials in each run. These subject-level averages (one per stimulus category) were standardized by applying z-score transformation with respect to the pre-stimulus baseline (−500 to −1 ms), and were projected onto the ICBM152 template (Fonov et al., 2009) for further group analysis.

### 2.6. Group statistical analysis and regions of interest (ROI)

We used two-tailed independent-samples permutation *t*-test with 1,000 randomizations to contrast z-scored brain activation maps (in ICBM152 template space) between obese and normal-weight individuals separately for each stimulus category. Brain regions showing statistically significant differences at *P* < 0.05 (false discovery rate [FDR] corrected) for a minimum duration of 20 ms were defined as regions of interest (ROI). The MNI coordinates of the ROI centroids, and their extents (number of source grid point within the ROI) and corresponding significant latency ranges were identified and extracted using an in-house developed Brainstorm plugin (Tables 1, 2). The ROIs were labeled based on the AAL3 atlas (Rolls et al., 2020) and their putatively associated brain networks [reward (Berridge and Kringelbach, 2015), cognitive control (Miller and Cohen, 2001; Ridderinkhof et al., 2004), attention (Corbetta and Shulman, 2002), and vision (Tootell et al., 2003)] were determined from established literature.

**TABLE 1:** Significant differences between obese and normal-weight individuals for high-calorie food images.

ROI	Network	MNI coordinates	No. points	Latencies (ms)
**Obese < normal-weight**				
sgACC L	Reward	−3, 32, −5	23	139–563
sgACC R	Reward	7, 35, −2	16	139–733
VMPFC R	Reward	10, 41, −7	11	143–485
aOFC R	Reward	31, 36, −16	37	156–637, 799–978
IFGorb R	Control	32, 32, −10	16	171–637, 799–820
Caudate R	Reward	17, 21, −2	23	172–586
VMPFC L	Reward	−9, 34, −12	11	186–724
mOFC L	Reward	−14, 25, −20	62	191–733
Caudate L	Reward	−12, 16, 0	50	266–821, 962–1000
NAc L	Reward	−8, 15, −8	19	269–733
Olfactory cortex L		−7, 17, −11	25	269–733
PHG L		−25, −10, −33	13	288–896
Olfactory cortex R		6, 25, −3	6	290–408
NAc R	Reward	11, 20, −4	11	324–424
IFGorb L	Control	−24, 30, −9	15	340–815, 971–1000
TP L		−24, 11, −37	12	344–673
Amygdala L	Reward	−24, 1, −22	11	360–1000
MFG L	Control	−33, 47, 9	29	433–1000
Insula L	Reward	−25, 28, 3	7	442–721
**Obese > normal-weight**				
MCC		3, 8, 44	18	79–121, 207–272
Calcarine cortex	Vision	5, −87, −7	32	91 – 125
IPL L	Attention	−31, −54, 35	9	157 – 254
MOG L	Vision	−26, −89, 7	27	168 – 310
Lingual gyrus R	Vision	20, −69, −9	14	211 – 280

Regions of interest (ROI) with significantly reduced (obese < normal-weight) and elevated (obese > normal-weight) responses in obese versus normal-weight individuals, their putatively associated brain networks, MNI coordinates of centroids, number of source grid points and significant latency ranges are shown. L, left; R, right; sgACC, subgenual anterior cingulate cortex; VMPFC, ventromedial prefrontal cortex; aOFC, anterior orbitofrontal cortex (OFC); IFGorb, inferior frontal gyrus pars orbitalis; mOFC, medial OFC; NAc, nucleus accumbens; PHG, parahippocampal gyrus; TP, temporal pole; MFG, middle frontal gyrus; MCC, midcingulate cortex; IPL, inferior parietal lobule; MOG, middle occipital gyrus.

**TABLE 2:** Significant differences between obese and normal-weight individuals for low-calorie food images.

ROI	Network	MNI coordinates	No. points	Latencies (ms)
**Obese < normal-weight**				
pOFC L	Reward	−19, 18, −21	45	365–1000
Putamen L	Reward	−15, 14, −4	34	406–1000
NAc L	Reward	−10, 14, −8	12	508–1000
Olfactory cortex L		−16, 9, −16	16	406–1000
Insula L	Reward	−25, 13, −19	9	406–1000
PHG L		−22, −4, −30	28	416–1000
TP L		−27, 9, −34	57	402–1000
Amygdala L	Reward	−21, 1, −20	13	417–1000
Precuneus R		9, −62, 52	12	607–1000
**Obese > normal-weight**				
Calcarine cortex	Vision	3, −89, −10	37	91–130
MOG L	Vision	−26, −89, 7	27	168–310
Fusiform gyrus R	Vision	27, −75, −13	13	415–1000
IPL L	Attention	39, −51, 43	20	305–501
MCC R		−8, −29, 43	17	79–116

pOFC, posterior OFC. Remaining conventions as in Table 1.

### 2.7. Regression analysis

To investigate whether pretreatment brain responses to images of food can predict weight change after 6 months in treatment, multiple regression analysis was performed on patient data. The ROIs were first merged into larger areas according to their associated brain network (reward, cognitive control, attention and vision), and the mean brain activations across each of these network-specific areas in the 50–150, 150–300, and 300–1000 ms post-stimulus periods was calculated. We selected these specific time periods (50–150, 150–300, and 300–1000 ms), as it is recognized that brain responses occurring before 150 ms reflect automatic preconscious information processing, and activations occurring after 300 ms include controlled conscious level processing, while the presence of conscious processing in the 150–300 ms latency range is currently debated (Dehaene and Changeux, 2011; Koivisto and Grassini, 2016; Sergent, 2018; Förster et al., 2020).

A mixed-effect linear regression model was constructed including the BMI percentage change as the dependent variable, the brain activation in each network and time period (12 variables), food stimulus category (high-calorie versus low-calorie food) and pretreatment BMI as the fixed-effect independent variables, and the subject as the random-effect independent variable (random intercept). A stepwise backward elimination approach minimizing the Akaike information criterion (AIC) was then used to find the best-fit model. Variance inflation factors (VIF) and correlation matrix plots (Supplementary Figure 1) were used to detect possible multicollinearity between the independent variables. To evaluate significance of fixed-effects in the best-fit model, analysis of variance (ANOVA) with χ2 test was used to compare it with a null model, which included only the random intercept.

### 2.8. ROI temporal dynamics

To explore the precise temporal dynamics of the identified ROIs, their activation time courses were generated by averaging, for each time sample (−500–1000 ms with a step of 1 ms), the activations (z-score values) across source grid points within the ROI (scouts time series with Mean scout function in Brainstorm). The ROI time courses were generated separately for each subject and stimulus category, and were then averaged across subjects in each group (obese and normal-weight). The time courses were baseline corrected (−500 to −1 ms) and smoothed with a 5 ms running window for illustrative purposes.

### 2.9. Statistical analysis of weight change data

Generalized linear model was used to evaluate associations of the degree of weight change with the subject group (obese versus normal-weight), gender (male versus female), food preference (high-calorie versus low-calorie) and pretreatment BMI. χ2 test was used to assess the association of subject group with the preferred food category and portion size. Fisher’s exact test was used when the crosstabulation contained a cell with a frequency of less than five. Statistical analyses were performed using R (R Core Team, 2022).

## 3. Results

### 3.1. Weight change data

**FIGURE 2:**
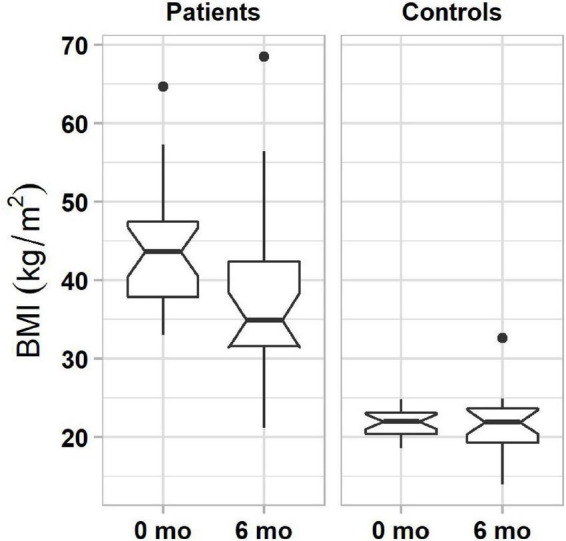
Boxplots of BMIs in obese **(left column)** and normal-weight **(right column)** individuals before and 6 months after the start of lifestyle weight-loss intervention. In each plot, boxes represent the interquartile ranges (IQR) with the lower and upper hinges corresponding to the first and third quartiles, and the line inside the box indicating the median; whiskers represent the lowest and highest values within 1.5 × IQR of the hinges (the first or third quartiles); black dots represent outlying values beyond the whiskers. The notches show the 95% confidence interval for the medians (1.58 × IQR/$\sqrt{n}$).

Results of generalized linear model analysis showed only a significant main effect of subject group (β = 4.7, *t*(33) = 2.17, *P* = 0.037). Patients, who pursued lifestyle changes, lost an average of 5.89% of their initial body weight in 6 months, significantly more than controls (0.45%, Figure 2). Fifty-four percent of patients (13 of 24) lost more than 5% weight, and no patient gained 5% or more weight. In the control group, 8% (2 of 24) lost and 8% (2 of 24) gained more than 5% weight.

The post-experimental assessment of each participant’s preference for high- or low-calorie food revealed that more controls than patients preferred high- than low-calorie food (62% versus 21%, χ^2^ = 9.5, *P* = 0.001). There was no significant group difference between the preferred portions sizes (*P* = 0.61), with 87% of patients and 87% controls selecting a full portion. However, see also the section “4.3. Limitations.”

### 3.2. MEG data

#### 3.2.1. Differences in brain activity between obese and normal-weight individuals

**FIGURE 3:**
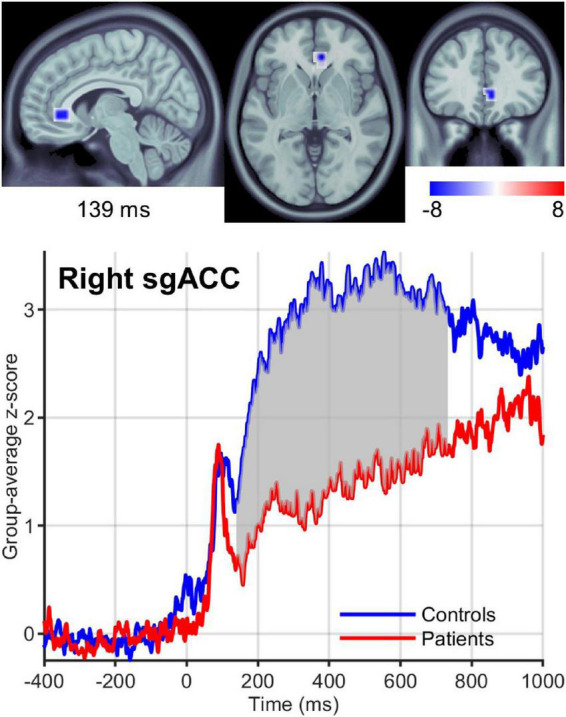
Right sgACC response to high-calorie food images. **(Upper row)** Brain region showing the earliest significantly (*P* < 0.05) reduced response in the reward system in obese versus normal-weight individuals at 139 ms post-stimulus. **(Lower row)** Group-averaged activation time courses of the sgACC R ROI. Gray shaded area indicates time period with significant difference between obese and normal-weight individuals.

**FIGURE 4:**
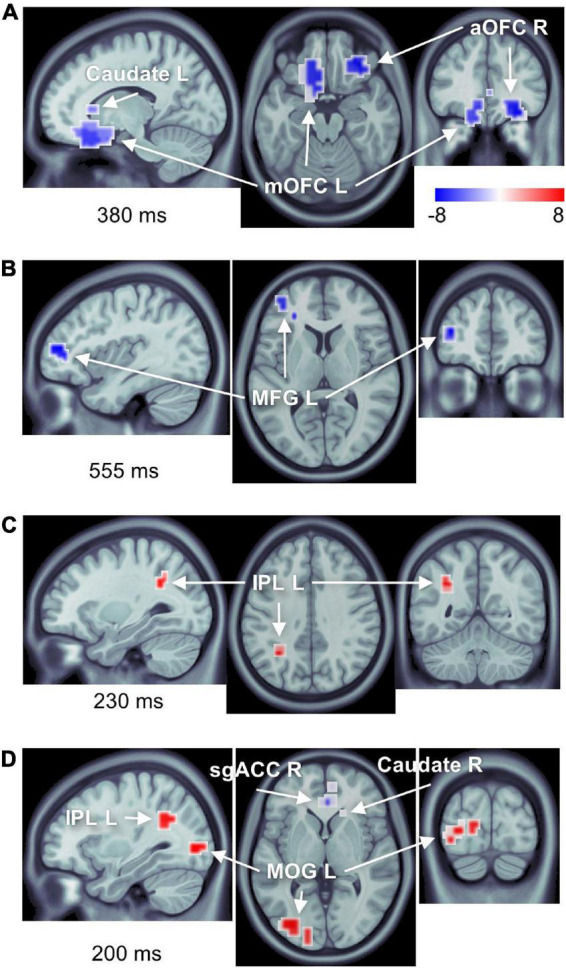
Number of brain regions showing differential responses to high-calorie food images in obese versus normal-weight individuals. Complete list of ROIs is provided in Tables 1, 2. Activations are shown at the peak latency of the principle ROI in each panel. **(A)** Reduced responses within the reward system (mOFC L, aOFC R, and caudate L). **(B)** Reduced response within the cognitive control network (MFG L). **(C)** Elevated response in the attentional control region (IPL L). **(D)** Elevated response within the visual cortex (MOG L), as well as elevated response in IPL L, and reduced responses in sgACC R and caudate R.

We found significant differences in brain activity between obese and normal-weight individuals (*P* < 0.05, FDR-corrected) for high- and low-calorie food stimuli (Tables 1, 2), but not for nonfood stimuli. The differences elicited by high-calorie food images were observed within brain networks associated with reward processing (Berridge and Kringelbach, 2015), cognitive control (Miller and Cohen, 2001; Ridderinkhof et al., 2004), attention (Corbetta and Shulman, 2002) and visual processing (Tootell et al., 2003). Reward system reactivity was lower in obese than normal-weight individuals beginning at around 100 ms after stimulus onset, and reaching statistical significance at 139 ms in bilateral subgenual ACC (sgACC, Figure 3) and at 143 ms in right VMPFC. The most extensive difference was found in the left medial OFC (mOFC) between 191 and 733 ms post-stimulus (Figure 4A). In addition to the reward system, significantly reduced responses in obese were found in the cognitive control network (Ridderinkhof et al., 2004; Niendam et al., 2012), such as in left middle frontal gyrus (MFG, Figure 4B) and bilateral IFG pars orbitalis (IFGorb). Greater reactivity in obese than normal-weight individuals was observed in brain regions of attentional control (Figure 4C) and visual processing (Figure 4D).

**FIGURE 5:**
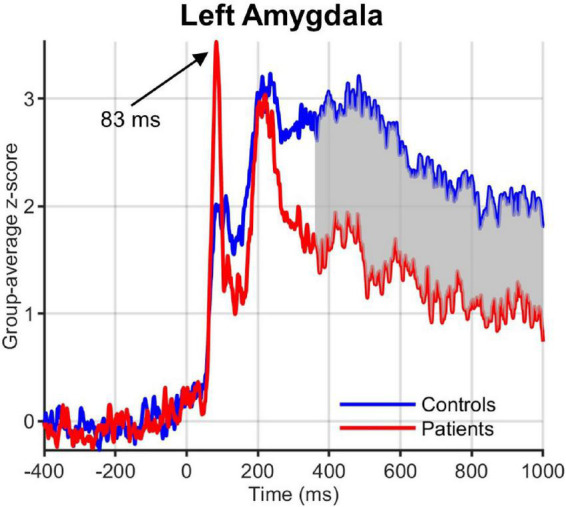
Group-averaged activation time courses of the amygdala L ROI. Gray shaded area indicates time period with significant difference between obese and normal-weight individuals. Note the early peak at 83 ms that was greater in obese than normal-weight individuals, but was not statistically significant.

Visual inspection of the ROI time courses revealed an earlier transient with a peak at ∼90 ms after stimulus onset, which was strongest in the left hemisphere subcortical structures, including amygdala (Figure 5) and striatum (caudate and NAc). While this early peak was higher in obese than normal-weight individuals in a number of ROIs (left amygdala, caudate, mOFC and bilateral NAc), the differences were not significant according to our criteria (FDR-corrected *P* < 0.05 and minimum duration of 20 ms).

Neural reactivity to low-calorie food images was significantly lower in obese than normal-weight individuals in the reward system, and was greater in brain regions of attentional control and visual processing (Table 2). Visual inspection of the ROI time courses showed that most ROIs and latencies identified for high-calorie food images were similarly affected also in response to low-calorie food images; however, here the obesity-related effects reached statistical significance (*P* < 0.05, FDR-corrected) only in a subset of regions and latencies. Two notable differences were evident. First, no significant alteration was identified in the cognitive control network in response to low-calorie food images. Second, reward system activity was significantly altered only in the late, controlled stage of processing (300–1000 ms). The only additional difference in response to low-calorie food images was found in a small region in the right precuneus area at later latencies (607–1000 ms).

#### 3.2.2. Pretreatment brain activity predicting weight loss after 6 months in treatment

**FIGURE 6:**
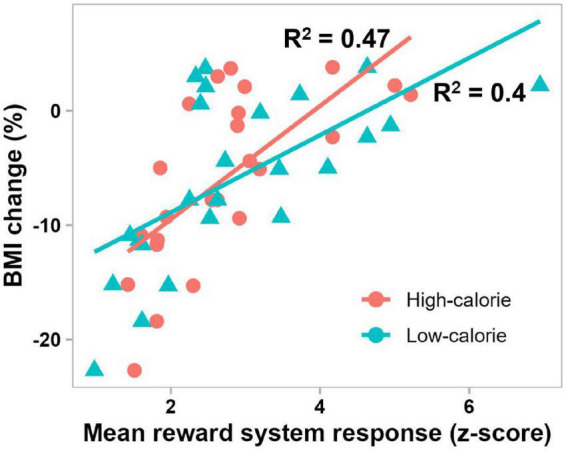
Scatterplot showing significant correlations between BMI change after 6 months in treatment and mean pretreatment reward system response to high-calorie (red) and low-calorie (green) food images in the 300–1000 ms period (*P* = 0.0002 and *P* = 0.0009, respectively).

The best-fit mixed-effect regression model, obtained by stepwise backward elimination approach, included fixed effects of brain activations in the reward system in the 300–1000 ms period, cognitive control network in the 300–1000 ms period, attentional control region in the 50–150 ms and 300–1000 ms periods, and visual cortex in the 50–150 ms period, and random effect of subject. The other variables including remaining brain activations (7 of 12 variables), food stimulus category and pretreatment BMI were eliminated by the stepwise backward approach. Of note, all activations in the 150–300 ms period were eliminated. This model performed significantly better than the random intercept-only null model (χ^2^(5) = 96.36, *P* < 0.0001), and showed significant main effects of the reward system (β = 0.91, *t*(19.76) = 8.16, *P* < 0.0001), cognitive control network (β = −0.43, *t*(19.76) = −3.9, *P* = 0.0009) and attentional control region (β = 0.37, *t*(19.76) = 4.03, *P* = 0.0007) in the 300–1000 ms period, and only attentional control region in the 50–150 ms period (β = 0.28, *t*(19.76) = 2.68, *P* = 0.015). For all variables, the VIF was less than 5, which together with the correlation matrix plots (Supplementary Figure 1), indicates that there was no significant collinearity between the independent variables. Follow-up simple linear regression analyses confirmed the direct effect of each of these significant variables (activations in reward system and cognitive control network in the 300–1000 ms period, and attentional control region in the 50–150 and 300–1000 ms periods) on BMI percentage change, with 0.22 ≤ | R^2^| ≤ 0.47, and *P* < 0.05. Figure 6 shows the significant relationship between reward system reactivity to food cues in the 300–1000 ms period and BMI change after 6 months in treatment, in accord with our second hypothesis.

## 4. Discussion

In the present study, we have identified, for the first time with high temporal resolution, the large-scale dynamics of brain reactivity to food cues in obese versus normal-weight individuals. Distributed sets of brain regions associated with the brain networks of reward (Berridge and Kringelbach, 2015), cognitive control (Miller and Cohen, 2001; Ridderinkhof et al., 2004), attention (Corbetta and Shulman, 2002) and visual processing (Tootell et al., 2003) displayed altered responses in obesity, in good agreement with earlier fMRI studies (Brooks et al., 2013; García-García et al., 2013; Pursey et al., 2014). Furthermore, pretreatment neural responses in the reward system, cognitive control network and attentional control region significantly predicted the outcome of the lifestyle weight loss intervention. As hypothesized, the brain responses to high-calorie food images in the reward system were altered in obesity beginning from the early, automatic stage of information processing (<150 ms). Consistent with our second hypothesis, the magnitude of food cue reactivity in the reward system significantly predicted the degree of weight loss after 6 months in treatment.

Our results are broadly consistent with the main tenets of the current neurocognitive models of obesity (Nederkoorn et al., 2006, 2010; Stice and Yokum, 2016; Lowe et al., 2019; Stice and Burger, 2019) in that we found that future weight change is positively correlated with food cue reactivity in the reward system and attentional control region (Stice and Yokum, 2016; Stice and Burger, 2019), and negative correlation with reactivity in the cognitive control network (Nederkoorn et al., 2006, 2010). Furthermore, neural responses elicited by food cues in obese versus normal-weight individuals were stronger in attention-related brain regions and weaker in the cognitive control network. However, our results are inconsistent with the assumed consequence of the incentive sensitization theory of obesity (Stice and Yokum, 2016; Stice and Burger, 2019) in that in the current study, food cue reactivity in the rewards system was reduced rather than elevated in obese versus normal-weight individuals. In sum, our findings are largely consistent with the theory that obesogenic behavior is mediated by increased attention and reward sensitivity to food cues, and reduced cognitive control over these processes. The current neurocognitive models are based on findings from fMRI studies, and therefore are unable to take advantage of the precise timing of neural activity. Our findings provide the temporal dynamics as well as the neural correlates of food cue processing, and can be utilized to extend and refine the current models.

### 4.1. Altered brain responses in obesity

#### 4.1.1. Hypoactivity in the reward system

We found lower reward system reactivity to food cues in obese than normal-weight individuals beginning at 139 ms post-stimulus. To our knowledge, this is the first study showing altered neural responses in the reward system in the early, automatic stage of information processing. This early altered reward response will likely have a widespread impact on ensuing neural processes, greatly affecting many aspects of brain function, including mood and choice behavior. Individuals with obesity may so require additional efforts and special cognitive strategies or medical treatment to regulate these automatic and controlled processes, and eating behavior. We suggest that to be effective, therapies should consider this aspect and design interventions that can target automatic reward processing.

The vast majority of neuroimaging studies of obesity and reward have used fMRI to investigate the reward system reactivity. However, due to the inherently poor temporal resolution of fMRI, it has not been possible to differentiate between early, automatic and late, controlled neural processes or examine the precise timing of reward processing in the brain. MEG and electroencephalography (EEG), which provide superior temporal resolution, could be used to address these shortcomings. However, previous MEG (Tschritter et al., 2006; Dubbelink et al., 2008; Stingl et al., 2010, 2012; Guthoff et al., 2011) and EEG (Nijs et al., 2008, 2010a,b; Hofmann et al., 2015; Hume et al., 2015; Reyes et al., 2015; Carbine et al., 2018; Chen et al., 2018; Iceta et al., 2020; Wang et al., 2022) studies have focused primarily on other aspects than the timing of neural reward processing. To the best of our knowledge, this is the first study examining the dynamics of the reward system reactivity to food stimuli in obesity.

The reward system brain regions identified in the current study have often been associated with obesity by earlier neuroimaging studies (Rothemund et al., 2007; Stoeckel et al., 2008; Bruce et al., 2010; Martin et al., 2010; Dagher, 2012; Dimitropoulos et al., 2012). However, contrary to the most studies and the prevailing view in the literature (García-García et al., 2013; Pursey et al., 2014), we found that in response to food images, especially high-calorie food images, activity in these regions was reduced rather than elevated in obese compared with normal-weight individuals. Similar “unexpected” findings in satiated overweight women have been reported earlier by Frankort et al. (2012). Further, in line with our results, few studies have observed negative correlation between activity in brain regions of reward system and BMI in normal-weight individuals (Killgore and Yurgelun-Todd, 2005; Toepel et al., 2012). Evidence suggests that both elevated and reduced responsivity of reward system to food are associated with obesity, and that the direction of association may depend on individual’s genotype (Stice et al., 2008, 2010, 2015).

Since cognitive and motivational factors can modulate neural activity within the reward system (Berkman, 2018; Giuliani et al., 2018; Roefs et al., 2018), it is also possible that in our patients, the subconscious negative attitude toward food, induced by their goal of losing weight through improved diet, resulted in suppression of reward system reactivity to food images. In line with this reasoning, in the post-experimental assessment, patients preferred less high-calorie and more low-calorie food than normal-weight controls, which is in accord with earlier studies showing higher dietary restraints in overweight individuals (Frankort et al., 2012; Ramírez-Contreras et al., 2021). In the present study, we recruited patients with class 2 or 3 obesity (BMI > 35 kg/m^2^), while previous studies have typically used subjects with overweight or class 1 obesity. It is possible that different neural mechanisms underlie food cue reactivity in individuals with severe versus moderate obesity or overweight.

Few other alternative, albeit less likely, explanations for these divergent results exist. Previous studies have used blood oxygenation level-dependent (BOLD) fMRI to examine food reward processing, while we have used MEG in the present study. BOLD fMRI and MEG measure distinct physiological processes, hemodynamics and electrophysiology, at considerably different time scales, seconds and milliseconds, respectively. Their signals behave differently, especially for neuronal activity at lower frequencies (<50 Hz) where a negative correlation between them is observed (Zumer et al., 2010; Hall et al., 2014). Moreover, the relationship between BOLD fMRI and MEG signals can vary across brain regions, activation frequencies and tasks (Mukamel et al., 2005; Furey et al., 2006; Zumer et al., 2010; Conner et al., 2011; Kujala et al., 2014). Although fMRI may provide a more accurate spatial localization of activity in some brain areas, MEG provides a more direct measure of neural activity with a millisecond time resolution at which brain operates. Another explanation, suggested by Frankort et al. (2012), relates to differing experimental designs. In this and Frankort et al. (2012) studies lower neural reward reactivity was found in overweight versus normal-weight participants using event-related design, whereas in the other studies with the opposite findings, blocked design was employed. In blocked design, brain activity is measured in response to a group of stimuli. Event-related design, on the other hand, allows measurement of responses to individual stimuli, and therefore may provide more accurate results (Frankort et al., 2012).

#### 4.1.2. Hypoactivity in the cognitive control network

We found reduced reactivity to high-calorie food images in the cognitive control network (bilateral IFGorb and left MFG) in obese compared with normal-weight individuals, indicating diminished inhibitory control over food cue processing in obesity (Aron et al., 2004; Aron, 2007; Jahanshahi et al., 2015), in accord with earlier neuroimaging studies (Batterink et al., 2010; Nummenmaa et al., 2012; Giuliani et al., 2014; Tuulari et al., 2015; Janssen et al., 2017; Han et al., 2018). Thus individuals with obesity may fail to suppress the prepotent responses to food cues, leading them to crave and overeat (Batterink et al., 2010). The altered response in this network emerged at 171 ms, in the intermediate stage of processing (150–300 ms), in the right IFGorb, where it continued until 637 ms post-stimulus. The most consistent ERP finding in obesity research is that the amplitude of the fronto-central P300 component (positive-going waveform ∼300–600 ms post-stimulus) in tasks requiring inhibitory control is smaller in obese than normal-weight individuals (Babiloni et al., 2009; Reyes et al., 2015; Chen et al., 2018; Iceta et al., 2020; Wang et al., 2022). The brain source of this ERP component has been localized to the inferior frontal cortex (Enriquez-Geppert et al., 2010), in agreement with our findings.

#### 4.1.3. Hyperactivity in the visual and attentional control regions

Greater responses in obese than normal-weight individuals were observed in regions of visual cortex and attentional control. The altered visual processing began early, in the primary visual cortex at 91 ms post-stimulus and extended to extrastriate cortex in the ∼150–300 ms period, indicating an automatic attentional engagement and enhanced processing of food images in individuals with obesity. This attentional bias has often been associated with overeating and weight gain (Berridge et al., 2010; Nijs et al., 2010b; Werthmann et al., 2011, 2014). In accord with this result, ERP studies have found that the P200 component (positive-going waveform ∼200–300 ms), which is associated with visual and attentional processing, in response to food-related stimuli is significantly greater in overweight (Hume et al., 2015) and obese (Nijs et al., 2010a) than normal-weight individuals. Further, two behavioral experiments using eye-tracking and visual probe paradigms have shown that obese individuals automatically direct their attention to food-related stimuli (Castellanos et al., 2009; Nijs et al., 2010b), supporting our findings. However, recent meta-analysis of 19 studies showed that behavioral measures of attentional bias in visual probe, emotional Stroop and eye-tracking tasks did not differ between obese and normal-weight individuals, but ERP P200 response to food images was enhanced in individuals with obesity (Hagan et al., 2020). Thus, enhanced early neural processing of food images, supported by our findings, is currently the most reliable evidence in support of automatic attentional bias toward food stimuli in obesity.

#### 4.1.4. Hyperactivity in the midcingulate cortex

In this study, the earliest altered response to high-calorie food images in individuals with obesity was found at 79 ms post-stimulus in midcingulate cortex (MCC, or dorsal ACC), which is a key brain region involved in detection and resolution of cognitive conflicts (Botvinick et al., 2001, 2004; Ridderinkhof et al., 2004). It has been suggested that cognitive conflicts, indexed by MCC activity, are negatively reinforcing events (due to their high levels of information processing demands) that induce avoidance learning by suppressing reward reactivity to stimuli associated with the conflict (Botvinick, 2007). This account fits well with our findings. High-calorie food images induce greater cognitive conflict in obese individuals between the prepotent desire to eat and long-term goal of losing weight, which is reflected in significantly greater activity in MCC beginning at 79 ms post-stimulus. This response may then suppress food cue reactivity in the reward system, as seen in our data (beginning at 139 ms), to reinforce avoidance learning and behavior, in accord with patients’ motivational goals.

In ERP studies (Nieuwenhuis et al., 2003; Enriquez-Geppert et al., 2010), conflict detection and MCC activity have been associated with the fronto-central N200 component (negative-going waveform ∼200–300 ms). In line with our results (enhanced obesity-related MCC response in the 207–272 ms period), Chen et al. (2018) have found larger N200 amplitudes in a go/no-go paradigm in obese compared to normal-weight adolescents, and Watson and Garvey (2013) have found significant correlation between food-related N200 amplitude and BMI, but only in female participants. However, other studies did not find significant differences in N200 amplitudes between obese and normal-weight individuals (Reyes et al., 2015; Carbine et al., 2018; Wang et al., 2022).

#### 4.1.5. Comparison with previous ERP studies

A key contribution of the current study is the provision of temporal dynamics of altered regional brain activations in obesity, and their relation to processing stages. Hereof our results are largely consistent with a number of ERP studies that have found obesity-related alterations in the N200 (Watson and Garvey, 2013; Chen et al., 2018), P200 (Nijs et al., 2010a; Hume et al., 2015), and fronto-central P300 (Chen et al., 2018; Wang et al., 2022) components. While a clear correspondence between these ERP components and brain sources identified in our study may not be established, we speculatively suggest that they correspond to altered responses in MCC (207–272 ms), extrastriate visual areas (∼150–300 ms) and IFGorb (171–637 ms), respectively. Our results provide additional spatiotemporal details, which have not been obtained in earlier studies. We have identified larger network of dysfunctional brain regions than could be suggested from the previous ERP studies. Importantly, the earliest functional alterations identified here occur ∼100 ms earlier that the effects reported in previous studies. ERP signal analysis is substantially less sensitive than our MEG source analysis, and therefore such investigations have likely missed substantial spatiotemporal details, including the earliest obesity-related functional alterations. To our knowledge, this is the first report providing temporal dynamics of altered neural activity in an extended number of brain regions in obesity, and showing altered brain responses in obesity before 150 ms post-stimulus.

#### 4.1.6. Differences between responses to high- and low-calorie food images

More widespread and larger differences between obese and normal-weight individuals were found for high- than low-calorie food images. In contrast to high-calorie food images, statistically significant differences elicited by low-calorie food images did not involve cognitive control regions, or reward system activity in the early, automatic stage of processing. Based on these results and previous evidence showing that high- and low-calorie food stimuli may be processed by different networks in the brain (Killgore et al., 2003; Toepel et al., 2009), it is tempting to suggest that different brain networks and neural mechanisms are involved in altered processing of high- versus low-calorie food cues in obesity. Most notably differentiating between altered reward responses that emerged early in the automatic stage of processing for high-calorie food stimuli versus such responses that occurred only later, in the controlled stage of processing for low-calorie food images. Furthermore, neural processing of low-calorie food images in individuals with obesity may be properly regulated by cognitive control regions.

However, our exploratory analysis revealed that most brain regions and latencies that were affected in obesity in response to high-calorie food stimuli, including the early reward system activations, were qualitatively similarly affected also in response to low-calorie food images, even though the differences in many cases did not reach statistical significance. Thus it is also possible that the same network of brain regions mediates the altered processing of food cues in obesity, independent of food’s caloric content. In the case of low-calorie food images, the obesity-related effects may be small, possibly due to their less rewarding nature, such that we do not have the statistical power to distinguish them.

### 4.2. Pretreatment neural reactivity predicting outcome of weight loss intervention

Pretreatment neural responses to food cues significantly predicted the outcome of the lifestyle weight loss intervention. As expected from earlier empirical studies, future weight change was positively correlated with neural reactivity to food images in the reward system (Yokum et al., 2011, 2014; Demos et al., 2012; Murdaugh et al., 2012; Stice et al., 2015; Hermann et al., 2019) and attentional control region (Calitri et al., 2010; Nijs et al., 2010b; Murdaugh et al., 2012; Werthmann et al., 2014), and negatively correlated with reactivity in the cognitive control network (Kishinevsky et al., 2012; Weygandt et al., 2013; Neseliler et al., 2019). Thus lower reward and attention responsivity, and higher neural cognitive control during food cue processing at the start of the lifestyle weight loss intervention were predictive of successful weight loss after 6 months in treatment, in general agreement with the current neurocognitive models (Nederkoorn et al., 2010; Stice and Yokum, 2016).

Many neuroimaging studies have examined association between food cue reactivity and future weight gain (Stice et al., 2010, 2015; Yokum et al., 2011, 2014; Demos et al., 2012; Kishinevsky et al., 2012); however, relatively few studies have focused on neurofunctional predictors of treatment-related weight loss (Murdaugh et al., 2012; Weygandt et al., 2013; Hermann et al., 2019), reporting some consistent, but also conflicting results. In accord with our results, Murdaugh et al. (2012) have found that pretreatment neural reactivity to high-calorie food images in brain regions of the reward system (e.g., ACC, insula) and attentional control (e.g., IPL) is positively correlated with weight change after 12 weeks in a lifestyle weight loss program. However, they did not find association between activity in cognitive control regions (e.g., IFG) and the treatment outcome, which is in contrast to our findings and those of Weygandt et al. (2013). Weygandt et al. (2013) have found that weight change after 12 weeks of dieting was positively correlated with pretreatment neural reactivity in insula (part of the reward system) and negatively correlated with reactivity in DLPFC (part of cognitive control network), which are concordant with our findings. However, in contrast to our results and those of Murdaugh et al. (2012), they have found also a negative correlation between the weight change and activity in VMPFC, which is part of the reward system. Hermann et al. (2019) found no relationship between pretreatment neural responses and weight change after 6-month intervention. However, they found that changes in striatum reactivity between the pretreatment and 1 month in treatment could significantly predict treatment outcome. Diet-induced early changes in activity of the cognitive control network have also been shown to predict weight loss (Neseliler et al., 2019).

Our result regarding the predictive role of reward system reactivity in weight loss is generally consistent with and complements the results of recent neuroimaging studies showing that increased reward reactivity to food cues predicts future weight gain (Yokum et al., 2011, 2014; Demos et al., 2012; Stice et al., 2015). In line with these findings, increased activity in brain reward regions has been associated with hunger and craving (Del Parigi et al., 2002; Siep et al., 2012; Frankort et al., 2014; Miedl et al., 2018), which are counterproductive for weight loss and promote weight gain. It is possible that our patients with higher reward reactivity experienced greater food craving when encountered such cues, which could have contributed to their relative lack of success in losing weight. Although responses in brain reward regions were altered in obesity in all investigated time periods beginning from the early, automatic stage of processing, only the activity in the 300–1000 ms period showed significant association with weight change, which may suggest that conscious downregulation of food reward reactivity plays an important role in successful weight loss through lifestyle intervention.

IPL response in the 50–150 ms time period was the earliest neural activity significantly associated with the degree of weight change. This area is a key node in the frontoparietal attentional network that is involved in directing attention toward motivationally salient stimuli (Behrmann et al., 2004; Ptak, 2012; Igelström and Graziano, 2017). Responses in IPL related to selective attention have been identified in various tasks as early as 40 ms after stimulus onset (Poghosyan and Ioannides, 2008; Burra et al., 2016), suggesting that this region can affect visual stimulus processing already at the very early, automatic stage of neural processing. Regarding visual food stimuli, directing attention to their hedonic value versus visual features has been shown to enhance reactivity in bilateral IPL (Franssen et al., 2020).

Behavioral studies have shown that food cues automatically capture attention, with hungry individuals exhibiting greater attentional bias (Mogg et al., 1998; Piech et al., 2010). Furthermore, significant correlation has been found between selective attention to food cues and subsequent food intake (Overduin et al., 1995; Kakoschke et al., 2014; Kemps et al., 2014; Werthmann et al., 2014). An association between attentional bias toward food cues and reward system reactivity has also been suggested (Siep et al., 2009; Pohl et al., 2017; Franssen et al., 2020). Accordingly, patients with elevated IPL response to food cues may have a greater automatic attentional bias, which could contribute directly, or indirectly through modulation of reward regions, to their difficulty in losing weight. This suggests that early differences in attentional processing can have significant behavioral and health effects.

Consistent with our finding on the predictive role of cognitive control regions, such as inferior and middle frontal gyri, several studies have found that activity in these regions during a delay discounting task is positively correlated with weight loss maintenance (Weygandt et al., 2015) and negatively correlated with weight gain (Kishinevsky et al., 2012). Furthermore, reduced activity in DLPFC and IFG in response to high-calorie food images has been associated with increased food intake (Cornier et al., 2010; Tryon et al., 2013; Lopez et al., 2014, 2016). It is evident from these and our findings that greater recruitment of cognitive control regions during food cue processing promotes anti-obesogenic behavior, likely through inhibitory influence or balanced interaction with the reward system (Jahanshahi et al., 2015; Lopez et al., 2017; Lowe et al., 2019). Similar to the reward system, only activity in the 300–1000 ms time period was significantly associated with future weight change, which may suggest that conscious cognitive regulation is necessary for successful weight loss through lifestyle changes.

In summary, our results suggest neural markers that may predict outcome of lifestyle weight loss intervention, which are well consistent with the known neural vulnerability factors for obesity and weight gain (Stice and Yokum, 2016; Stice and Burger, 2019), as well as with the reward-centered (Stice and Yokum, 2016; Stice and Burger, 2019), cognitive control-centered (Lowe et al., 2019) and balance-based models (Lopez et al., 2017) of obesity. They provide additional support to some of the predictive markers suggested by earlier fMRI studies (Murdaugh et al., 2012; Weygandt et al., 2013) and add crucial information on processing stages of predictive neural responses. The pattern of brain activations presented here suggests that lowered sensitivity to food cues as well as heightened self-control may be necessary for successful weight loss through lifestyle changes.

### 4.3. Limitations

The present study has several limitations. First, in our experimental design, we did not consider or control for the exact mental processes in which the participants were engaged in during the passive viewing of stimuli. This limits our ability to definitely associate altered neural activity with specific cognitive processes or interpret the results in such terms. Second, the food preferences obtained in post-experimental inquiry could be misleading. Evidence suggests that social norms influence food choice (Nook and Zaki, 2015). Hence, after the experiments, our participants could have chosen the foods in line with the social norms rather than subjective preference. This alternative explanation nevertheless does not affect our main findings. Third, majority of our participants (18 of 24) were women. While having a large proportion of women participants is a common practice in most such studies, e.g., (Murdaugh et al., 2012; Weygandt et al., 2013; Hermann et al., 2019), there are known gender differences in brain activations associated with food cue processing and obesity (Cornier et al., 2010; Killgore and Yurgelun-Todd, 2010; Geliebter et al., 2013; Sala et al., 2019). Women typically exhibit greater reactivity to high-calorie food images in many of the brain regions identified in the current study, including reward and cognitive control regions. Fourth, we examined participants in a fasted state only. The neurofunctional differences between obese and normal-weight individuals could be different in fasted versus satiated state. Previous studies have reported that food cue reactivity in several regions of the reward system is typically greater in obese than normal-weight individuals in fasted than satiated state (Page et al., 2011; Martens et al., 2013). Since we observed reduced reward system reactivity in obese than normal-weight individuals, our findings should be valid also for the satiated state. Nevertheless, it would be informative to study the same groups of participants in both states to better understand the effect of satiety on neural processing of food cues in obese and normal-weight individuals. Finally, we have identified a distributed set of brain regions and their temporal dynamics, but did not assess the functional connectivity between these regions or their network structure and dynamics.

## 5. Conclusion

The current study provides several novel findings. First is the detailed characterization of the temporal dynamics of altered regional brain activations during food cue processing in obesity. Second is the uncovering of altered automatic neural processes in obesity, including altered automatic responses to high-calorie food images in the reward system. This is the first report showing altered brain activations in obesity before 150 ms, and as early as 79 ms after food stimulus onset. Third is the identification of the pretreatment brain activation profiles in the reward system, attentional control region and cognitive control network, which are predictive of the outcome of lifestyle weight loss intervention. We corroborate and add to the findings of previous neuroimaging studies of obesity and weight change, showing that conflict monitoring, selective attention, reward valuation and inhibitory control are impaired in obesity, and that decreased attention and reward valuation, and increased cognitive control during food cue exposure promote anti-obesogenic behavior and weight loss.

These findings significantly advance our understanding of neural mechanisms affected in obesity and supporting anti-obesogenic behavior, and can have important implications for the development of targeted therapies. We suggest that integrated strategies targeting multiple brain systems governing reward processing, attentional and cognitive control could improve the treatment outcomes. In addition to tailoring established pharmacological and cognitive-behavioral therapies, promising new approaches, such as noninvasive brain stimulation and neurofeedback (Ioannides, 2018), may be used to directly target these brain systems (Dalton et al., 2017).

## Data availability statement

The datasets presented in this article are not readily available because of the Ethics Committee stipulations. The anonymized data may be made available upon request, with the approval of KFMC Ethics Committee. Requests to access the datasets should be directed to VP, vpoghosyan@kfmc.med.sa.

## Ethics statement

The studies involving human participants were reviewed and approved by the King Fahad Medical City Ethics Committee. The patients/participants provided their written informed consent to participate in this study.

## Author contributions

VP, SI, KA-A, and SA-M contributed to the conception and design of the study. VP supervised the work, contributed to the data analysis, performed the statistical analysis, and wrote the first draft of the manuscript. KA-A recruited, evaluated, and treated the patients. FA-M, TA-O, and WA-S performed the experiments, collected and curated the data, and contributed to the data analysis. All authors contributed to the manuscript revision, read, and approved the submitted version.

## Footnotes

1
https://www.nutritics.com

2
http://neuroimage.usc.edu/brainstorm
