# Supplementary material for: Spatiotemporal profile of altered neural reactivity to food images in obesity: Reward system is altered automatically and predicts efficacy of weight loss intervention
Source: Front Neurosci. 2023 Feb 8;17:948063. doi: 10.3389/fnins.2023.948063 (PMC9944082; doi:10.3389/fnins.2023.948063)
Supplement: Supplementary file 1 [file Image_1.PDF]

Supplementary Material

Supplementary Figures

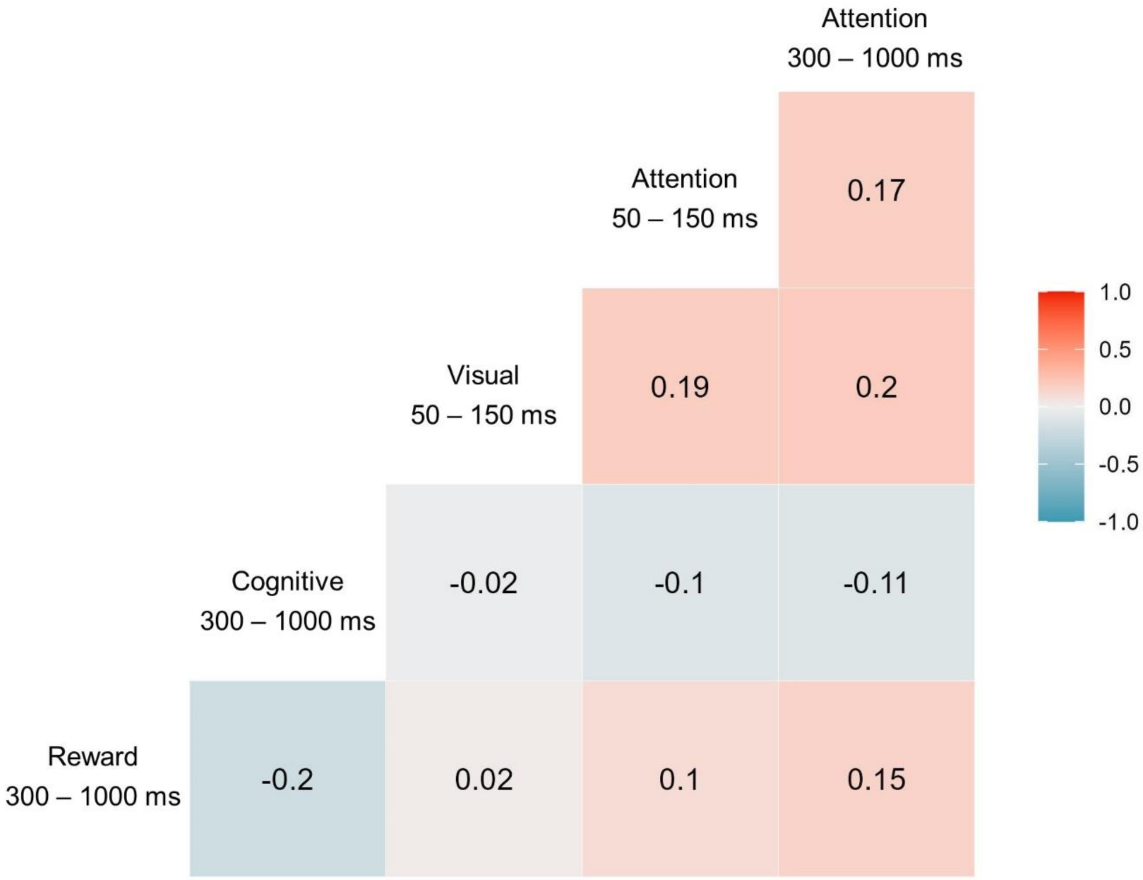

Supplementary Figure 1. Correlation matrix of best-fit regression model's independent variables. Correlation coefficients are color-coded and shown inside the corresponding cells.
